# Evidence of active sound production by a shark

**DOI:** 10.1098/rsos.242212

**Published:** 2025-03-26

**Authors:** Carolin Nieder, Eric Parmentier, Andrew G. Jeffs, Craig Radford

**Affiliations:** ^1^ Department of Biology, Woods Hole Oceanographic Institution, Woods Hole, MA, USA; ^2^ Morphologie Fonctionnelle et Evolutive, Université de Liège, Liège, Belgium; ^3^ Leigh Marine Laboratory, Institute of Marine Science, University of Auckland, Auckland, New Zealand; ^4^ Leigh Marine Laboratory, Institute of Marine Science, University of Auckland, Leigh, New Zealand

**Keywords:** elasmobranchs, *Mustelus lenticulatus*, rig, clicks, distress sound, startle response

## Abstract

Elasmobranchs are an evolutionarily ancient group of cartilaginous fishes that can hear underwater sounds but are not historically viewed as active sound producers. Three recent reports of several species of rays producing clicks in response to approaching divers have cast doubt on this long prevailing view and resulted in calls for more research into sound production in elasmobranchs. This study shows that the rig, *Mustelus lenticulatus*, produces clicks (mean SPL_rms_ = 156.3 dB re. 1 μPa ± 0.9 s.e.m. at approx. 30 cm) when handled underwater, representing the first documented case of deliberate sound production by a shark. Clicks were broadband (mean bandwidth = 23 kHz ± 0.1 s.e.m.), with peak energies between 2.4 and 18.5 kHz (mean peak frequency = 9.6 kHz ± 0.3 s.e.m.), and mean duration of 48.42 ms ± 2.9 s.e.m. Clicks contained considerably less energy in frequencies below 1 kHz, which overlap with the hearing range of the rig. We propose that forceful snapping of flattened teeth may be the sound producing mechanism based on the plated tooth morphology and the acoustic characteristics of these clicks. Further behavioural studies are needed to test whether clicks are incidental to the handling or a natural acoustic response of behavioural significance.

## 1. Introduction

Acoustic signals play a key role in intraspecific and interspecific communication in many marine organisms [1–3]. Over 1000 species of bony fishes produce sounds in a variety of social contexts, such as agonistic encounters, courtship, spawning and defence against predators [4–6]. By contrast, while elasmobranchs, including cartilaginous fishes such as sharks, skates and rays, possess auditory capabilities for hearing underwater sounds, they are not known to produce sounds voluntarily for communication [7–10]. Elasmobranchs have been shown to have some ability to produce sounds, incidentally, resulting from swimming [11,12], collision [13] and feeding [14,15]. However, published evidence that elasmobranchs are engaged in active sound production for means of communication is lacking [16,17].

This long-held view was challenged by a 2022 field study in Indonesia and Australia, which showed that two wild stingray species—the mangrove whipray (*Urogymnus granulatus*) and the cowtail stingray (*Pastinachus ater*)—produce clicks when approached by divers [18]. More recently, three batoid species in the Mediterranean Sea—the blonde ray (*Raja brachyura*) [19], the rough skate (*Raja radula*) and the marbled electric ray (*Torpedo marmorata*) [20]—were also reported to produce short, broadband clicks when disturbed by divers. In reporting these observations, the authors interpreted this acoustic behaviour as a response to a perceived disturbance or threat and called for more research into the ability of elasmobranchs to produce sounds [18–20]. While conducting behavioural hearing tests with several shark species, we observed that rigs, *Mustelus lenticulatus* Phillipps, 1932, consistently produce clicking sounds when they were handled underwater. In a similar manner, the ability to produce sounds in many, now well-known soniferous fish species was first discovered while hand-held in captivity or caught and handled in gill-nets [4,21,22].

Recordings of hand-held species allow for reliable comparisons between specimens and/or species because they can be recorded in standardized conditions [23–25]. Investigating active sound production in hand-held fish might sometimes seem limited because it is not always possible to determine whether the elicited sounds have any biological significance in the wild. Some fish produce the same type of sound in different behavioural contexts. For instance, the pennant bannerfish (*Heniochus chrysostomus*) produces the same type of calls when hand-held and when pursued by a conspecific in the field [26]. Similarly, piranhas [22] and holocentrids [27] produce the same sounds when hand-held and in other behavioural contexts (e.g. mobbing behaviour), supporting the idea that a sound could have different meanings in different behavioural contexts. These studies are highly valuable as a first step to explore acoustic behaviours of elusive species that are very difficult to observe in the wild, such as sharks.

The rig, also known as spotted estuary smoothhound, is a small, benthopelagic shark (70−150 cm) of the family Triakidae (hound sharks) [28]. The species is endemic to inner shelf areas, coastal waters (up to 1000 m depth) and shallow estuaries around New Zealand [28]. This mesopredatory shark mainly feeds on crustaceans, especially crabs on the sea floor [29] and is preyed upon by larger sharks, and marine mammals [30,31]. According to an anecdotal account by fish tag-and-recapture field researchers, juvenile rigs were heard to produce clicks in the wild while swimming in schools (Scott Tindale, Tindale Marine Research Charitable Trust, 2021, personal communication). Fish *et al.* [14] described loud noises, supposedly from the grinding of the flat granular teeth by a closely related species, the common smoothhound, *Mustelus mustelus*, that co-occurred during feeding [14].

Sound producing mechanisms are incredibly diverse in fishes [32,33]. The most important group of mechanisms involves the swim bladder and various types of sonic (drumming) muscles that induce swim bladder vibrations [34]. Stridulation, where hard structures such as bones and teeth are rubbed against each other, is a second important sound producing mechanism. Stridulation in fish can involve modifications of the pectoral girdle or fin (e.g. ridged pectoral spines in catfishes, Siluriformes) [35] or the grinding of pharyngeal teeth [10]. Elasmobranchs lack a swim bladder and are not known to possess any morphological specializations for sound production.

The goals of this study were to (i) describe the clicking behaviour of rigs when handled, (ii) characterize the temporal and spectral features of the clicks, and (iii) use morphological observations to propose a possible sound producing mechanism.

## 2. Material and methods

### 2.1. Animal collection and husbandry

Sound recordings were opportunistically obtained from 10 juvenile rigs (5 males, 5 females, TL range = 55.5−80.5 cm) while we conducted a behavioural acoustical conditioning study [36] from May 2021 to April 2022 (table 1). Seven of the rigs were caught in the Kaipara Harbour (New Zealand, North Island) and a further three rigs were obtained from local commercial fishermen who were also using baited hooks. Animals were housed at the Leigh Marine Laboratory in circular flow-through holding tanks (2.1 m diameter, 1.26 m water depth), supplied with filtered flow-through ambient seawater, and maintained on a mixed diet of squid and fish fed three times a week. The water temperature and salinity ranged from 14.7 to 22.5°C and 35 to 36 ppt, respectively. Sharks were acclimated for at least 1 week prior to experiments [36]. All procedures were conducted in accordance with ethics protocols 002066/AEC23071, approved by the University of Auckland’s Animal Ethics Committee (AEC).

**Table 1.** Size and sex of juvenile rigs and total number of clicks produced over a 20 s handling time used for analyses in this study.

ID	sex	total length (cm)	no. of clicks
1	male	73.5	5
2	female	55.5	3
3	male	69.5	10
4	female	61.5	5
5	male	80.5	10
6	male	65.0	15
7	female	70.0	16
8	female	67.0	9
9	male	62.0	16
10	female	56.8	4

### 2.2. Sound recordings and analyses

**Figure 1.**
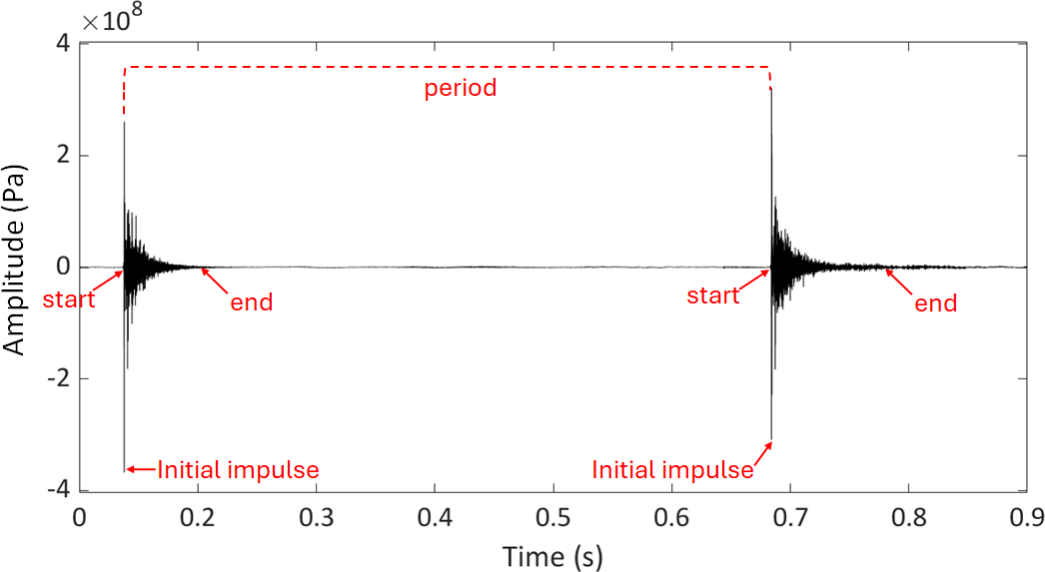
Waveform examples of two single-pulse clicks produced by rig when handled underwater. The click duration was determined by measuring the time difference between the start and the end of a click as shown by the arrows in red. The end of a click was measured where the tapering ‘tail’ blended in with the noise floor. The period was measured as the time difference between the maximum peaks of two subsequent clicks. The initial impulse is defined as the short onset portion that includes the maximum amplitude of the click.

All sound recordings were conducted in a rectangular plastic tank (4.8 m × 1.25 m, 0.76 m depth) with flow-through filtered sea water during our acoustical conditioning study [36]. A SoundTrap recorder (ST 300 HF, Ocean Instruments Ltd, New Zealand) was permanently set up inside the middle of the experimental tank at 30 cm below the water surface for calibration and monitoring of pure tone signals that were used for the conditioning training and testing procedures [36]. For all individuals, the hydrophone recorded continuously with a sampling rate of 48 kHz. Additional recordings were obtained for shark 6 using sampling rates of 44.1 kHz and 144 kHz and for shark 10 using a sampling rate of 144 kHz. As part of the routine conditioning and testing protocol, sharks were transferred from their holding tank into the experimental tank and identified based on their unique patterns of body markings. Handling took place in the experimental tank, with sharks submerged approximately at a 30 cm distance from the hydrophone. However, this is only an approximate estimate, as the sharks frequently moved during handling, causing small variations in angle and distance relative to the hydrophone and tank walls. During this brief handling time rigs were opportunistically observed to produce clicking sounds underwater. For each shark the continuous hydrophone recording for the 20 s from the start to end of handling were subsequently extracted and used for acoustic analyses. Raven Pro v.1.6 software was used to measure the duration (time between the defined start and end points of a click), period (time between two subsequent clicks), peak frequency (frequency of maximum power) and bandwidth (minimum to maximum frequency) of detected clicks (figure 1). In addition, the number of clicks produced during seconds ‘0−10’ and ‘11−20’ were counted. Plots of the waveform, power spectrum and spectrogram of representative clicks were generated using MATLAB.

### 2.3. Morphology of the cranial skeleton and teeth

It was thought that the teeth may be involved in producing the click sounds in rigs. Therefore, the gross morphology of cranial structures, including the positioning of the dental apparatus, was further investigated. The heads of two juvenile, male rigs (TL = 73.5 cm and 80.5 cm) were fixed in 10% buffered formalin and stored in 70% ethanol. To check for any obvious specialized sound producing cranial structures other than the teeth specimens underwent microCT scanning at the dynxlab platform (University of Antwerp) using a custom-made UnitimXL X-ray CT scanner (Tescan) operating at 65 kV and a current of 517 μA with a power of 30 W. This generated 2766 images and a voxel size of 64.6 μm. Three-dimensional (3D) images were produced in 16-bit and subsequently converted into 8-bit voxels using ImageJ. Amira 5.4.0 (VSG, FEI Company, Eindhoven, The Netherlands) was used for segmentation and 3D reconstruction of the chondrocranium, jaw cartilages and teeth. Shark heads were dissected, and teeth examined with a stereo dissection microscope (Leica, Wild M10, Wetzlar, Germany) coupled to a camera lucida and pictures were taken with a digital microscope (VHX-7000, (Keyence, Osaka, Japan).

### 2.4. Statistical analyses

A linear mixed-effects analysis was used to compare the number of clicks produced during seconds ‘0−10’ and ’11−20’ (electronic supplementary material, Data 1—Click.Timing.xlsx). Click counts were first square root-transformed due to zeros in the data and then fitted against a 2-level factor termed ‘seconds’ (‘0−10’; ‘11−20’) as the main effect and random intercepts for each shark subject.

Fish size and sex can affect sound characteristics [37,38]. Accordingly, the effects of total length and sex on the peak frequency, click duration and period were examined using linear mixed effects models [39]. Recordings of a ‘20 s handling period’ for each shark (*n* = 10) were used for analyses, yielding a total of 93 clicks (electronic supplementary material, Data 2—Click.Characteristics.csv) and 83 click periods (electronic supplementary material, Data 3—Click.Period.csv). To meet the model requirements, the click duration and period data were natural-log-transformed. Transformation was not required for the peak frequency data as their distribution was symmetrical. Each response variable (click duration, period, peak frequency) was fitted in a separate model against sex and total length as the main effects and random intercepts for each shark subject. Interactions between sex and total length were also investigated but found not to be significant for all models.

Additionally, we conducted fixed-effects analyses to test whether there are significant differences in click parameters (peak frequency, duration and period) between individual sharks (shark ID), where ‘shark ID’ was treated as a fixed effect.

All statistical tests were considered significant at *p* < 0.05. All statistical analyses were performed in R (v4.1.1) (R Core Team) using the ‘lm’ base function, ‘lmer’ from the ‘lme4’ package and ‘emmeans’ from the ‘emmeans’ package.

## 3. Results

### 3.1. Clicking behaviour

**Figure 2.**
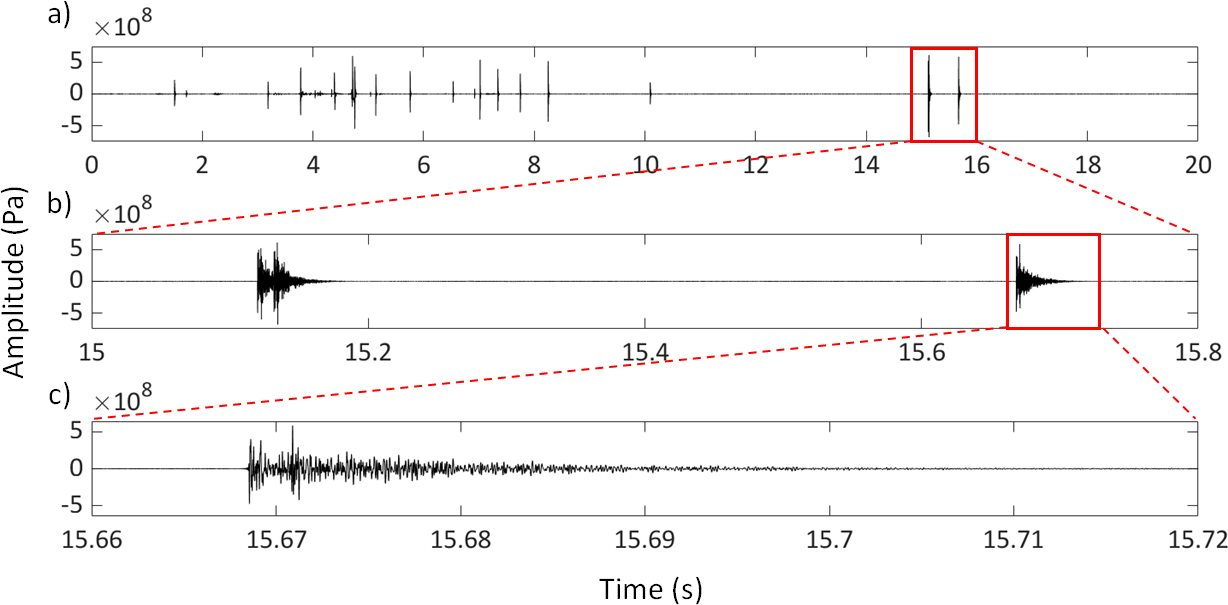
A series of clicks produced by a rig (*Mustelus lenticulatus*) during handling underwater. Waveforms of two selected clicks (b), marked by the red box in (a). Note the first is a double-pulse click (composed of two pulses that are fused together) and the second is a single-pulse click. Close-up view of the waveform of a single-pulse click (c), marked by the red box in (b).

**Figure 3.**
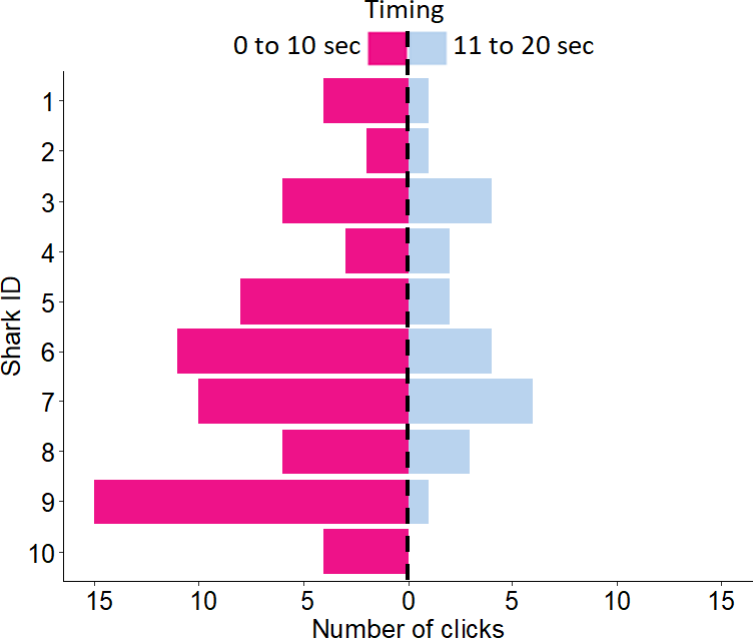
Number of clicks produced by 10 rigs (*Mustelus lenticulatus*) during handling for 20 s. On average the sharks produced significantly more clicks during the first 10 s than during the last 10 s (*p* = 0.0018).

On average the sharks produced nine clicks (± 2 s.e.m., range = 3–16) during handling underwater over the course of 20 s (figures 1 and 2a; Audio 1 and Audio 2 in electronic supplementary material). The sharks produced significantly more clicks during seconds ‘1−10’ (mean = 7 ± 1 s.e.m.) than during seconds ‘11−20’ (mean = 2 ± 1 s.e.m.) (*p* = 0.0018) (figure 3). Most of the clicks co-occurred with head and body movements, such as bending left to right. However, several clicks also occurred without any obvious head or body movements. Roughly 25% of the clicks co-occurred with an explosive sway (vigorous bending of the head and body from side to side), about 70% co-occurred with calm swaying (slow side to side movements), and 5% of clicks occurred in the absence of any obvious body movements. It is important to note that both explosive and calm swaying movements were not automatically accompanied by clicking sounds. For instance, during seconds ‘11−20’, when fewer clicks occurred, sharks continued with the same movements, while producing fewer or no more clicks compared with seconds ‘0−10’, when most clicks occurred. The click period was highly variable, ranging from 19 ms to 9.57 s, with a mean of 1.42 s (± 0.16 s.e.m.). There were no significant effects of sex (*p* = 0.208) or total length (*p* = 0.985) on the click period. The period did not differ significantly over the course of 20 s (*p* = 0.366).

The fixed effects model indicated a potential effect of shark ID on click period (*p* = 0.011). However, *post hoc* comparisons using Tukey’s method did not identify significant pairwise differences between individual sharks (figure 6). This lack of significance could stem from the limited sample size per individual shark (*n* = 2–15), which likely reduced the statistical power (power = 0.26) to detect true differences in click periods, even for medium effect sizes (*f* = 0.25).

### 3.2. Click characteristics

**Figure 4.**
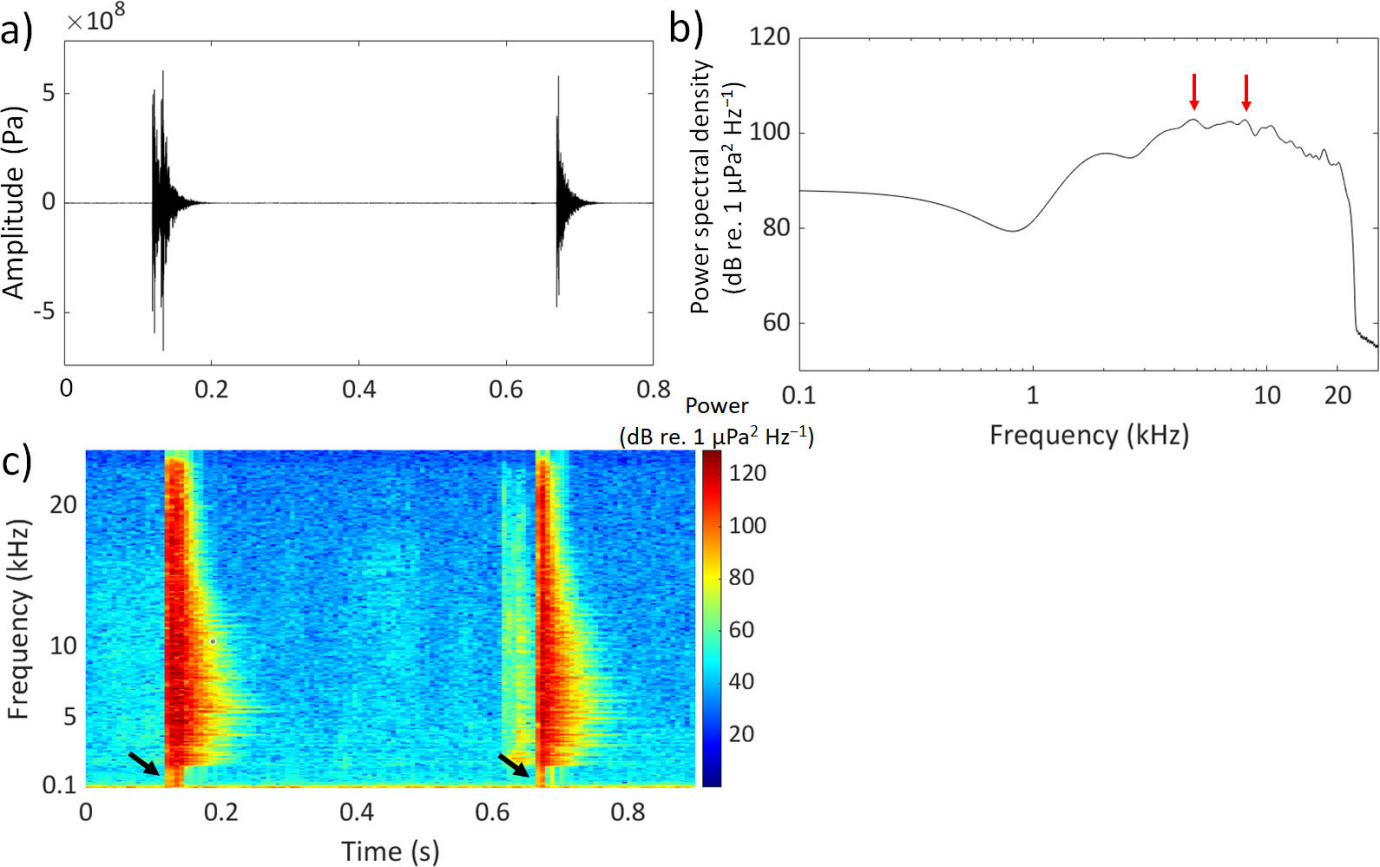
Temporal and spectral characteristics of two typical broadband clicks produced by the rig (*Mustelus lenticulatus*) during handling underwater in an experimental tank. Waveforms of a double-pulse and a single-pulse click (a). The power spectrum (b) shows there is significant energy between 2 and 20 kHz. The red arrows point to energy peaks at 5 kHz and 8 kHz in the signal, which drops off sharply at 20 kHz (smoothed using moving average filter, FFT = 144 kHz, window size = 256, overlap = 50%, sampling rate = 144 kHz). The spectrogram (c) shows that during the initial impulses of the clicks there are low frequency components (<1 kHz, indicated by the black arrows) that blend into the noise floor of the experimental tank (range = 0–200 Hz). These low frequency components contained in the initial impulses of the clicks overlap with the known hearing range of the rig and could potentially be detected by this species (FFT = 2048, window size = hamming(2048), overlap = 50%, sampling rate = 144 kHz).

Of the 93 clicks analysed, 74% consisted of a single pulse (‘single-pulse clicks’), while 26% comprised two fused pulses (‘double-pulse clicks’) (figures 2b and 4a). In double-pulse clicks, the average interval between the two pulses was 6.7 ± 1.5 ms (s.e.m.). The overall click duration varied within the range of 10.4−137.7 ms, with a mean duration of 48.42 ms (± 2.9 s.e.m.). The maximum sound pressure level of the clicks varied from 140.7 to 166.8 dB re. 1 μPa (mean max SPL_rms_ = 156.3 dB re. 1 μPa ± 0.9 s.e.m.) at approximately 30 cm from the animal. Each click was composed of a short initial impulse (range = 1.4−7 ms, mean duration = 3.9 ± 0.1 ms s.e.m.), followed by a longer tapering tail (figures 1 and 2b, c). The initial impulse was arbitrarily defined as the initial portion of the click that comprises the maximum amplitude and was measured from the beginning of each click to the approximate time point where the amplitude dropped sharply below the peak.

**Figure 5.**
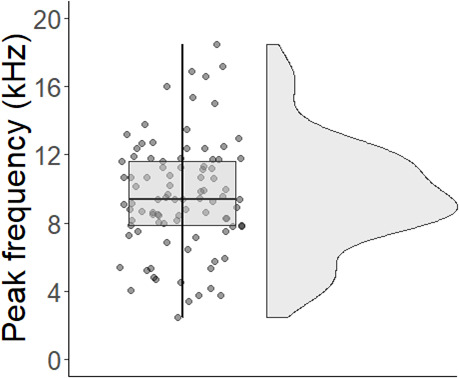
Distribution of peak frequencies of clicks produced by 10 rigs. Clicks (*n* = 93) contained significant energy in the frequency band from 2.4 to 18.5 kHz (mean = 9.6 kHz ± 0.3 s.e.m.). Most clicks (75%) had maximum energies of 7−14 kHz, and fewer of 2−6 kHz and 15−18 kHz.

A typical spectrum of a click was very broad with energies extending below 200 Hz and out to 24 kHz. The highest energies were contained within the frequency band of 2.4−18.5 kHz, with maximum energy peaks of 7−10 kHz (44% of clicks), 11−14 kHz (31%), 4−6 kHz (15 %), 15−18 kHz (8%) and 2−3 kHz (2%) (figures 4 and 5). The mean peak frequency was 9.56 kHz (± 0.34 s.e.m.). It was not possible to determine the lower frequency cut-off of the clicks because the lower frequency components (<1 kHz) of the initial impulse blended with the background sound of the experimental tank (range = 0−200 Hz) (figure 4c). Following the initial impulse, the remainder of the click was of lower frequency centred around 1.3 kHz (± 0.07 s.e.m.). The mean background sound level of the experimental tank was 109.9 SPL_rms_ dB re. 1 µPa (± 0.97 s.e.m.).

**Figure 6.**
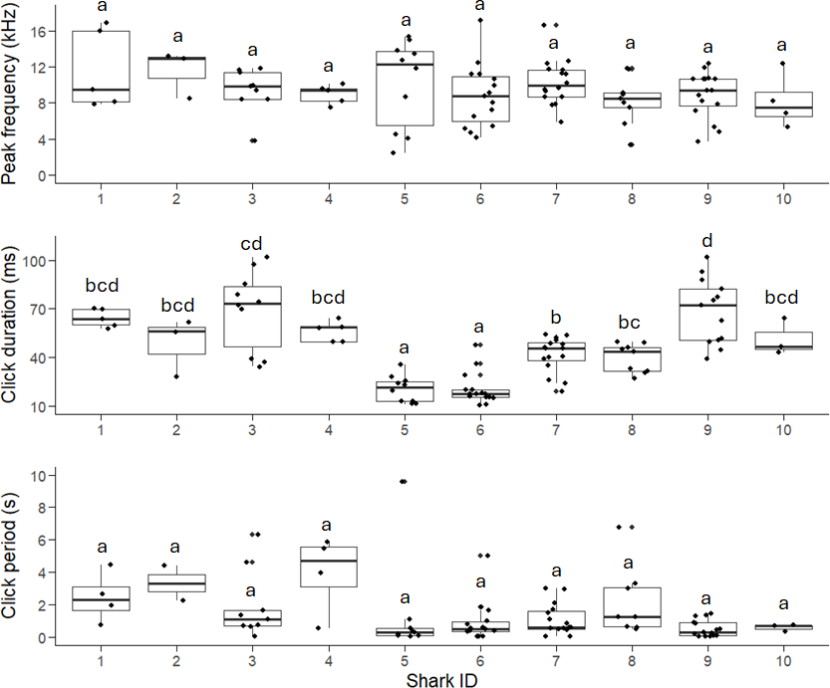
Distribution of peak frequency (a), duration (b), and click period (c) produced by ten rigs (Mustelus lenticulatus) during handling in an experimental tank. Lowercase letters indicate results of post hoc pairwise comparisons between individual sharks. Means not sharing a common letter are significantly different as determined by the Tukey test at P < 0.05).

There was no effect of sex on peak frequency (*p* = 0.681) and click duration (*p* = 0.693). Similarly, total length (range = 55.5−80.5 cm) had no effect on peak frequency (*p* = 0.182) and click duration (*p* = 0.280). Shark ID had no significant effect on peak frequency (*p* = 0.478). There was a significant effect of Shark ID on click duration (*p* < 0.001), with sharks 5 (male, TL = 80.5 cm) and 6 (male, TL = 65 cm) producing on average shorter clicks (range = 18.8−19 ms) than the remaining sharks (3 males, 5 females, TL range = 55.5–73.5 cm, mean click duration range = 38.6–73.1 ms) (figure 6). However, this result should be interpreted with caution, as click duration is likely affected by complex tank acoustics, including the animal’s angle and distance relative to the hydrophone and tank walls, where even minor variations can alter the duration of acoustic signals [40].

### 3.3. Cranial morphology and teeth structure

**Figure 7.**
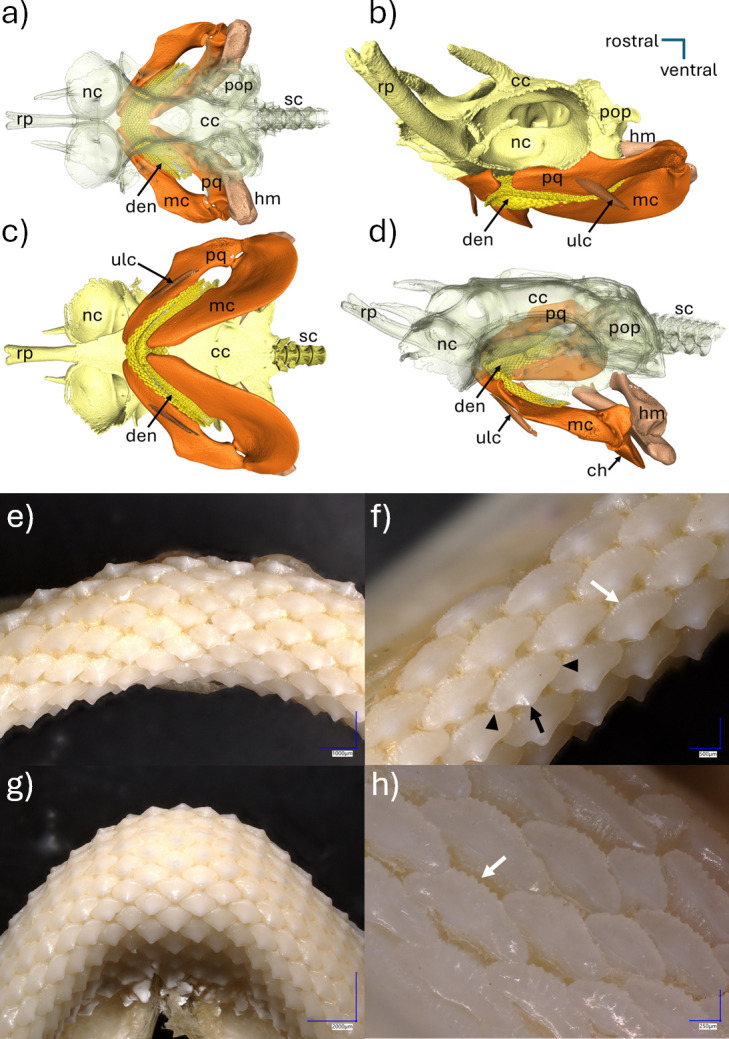
Cranial structures and details of the dentition in a male, juvenile rig (*Mustelus lenticulatus*). Dorsal (a), rostral (b), ventral (c), and lateral (d) views showing the locations of the cartilaginous braincase (chondrocranium), upper and lower jaw cartilages, and dental apparatus. Photographs taken of upper (e,f) and lower (g,h) jaw, showing blunt plated teeth with low rounded crowns and two weak lateral cusplets (f, black arrowheads). A peg extends from the lingual face of the crown (f, black arrow) and several short ridges are at the base of crown (f,h, white arrow). Abbreviations: cc, chondrocranium; ch, ceratohyal; den, dental apparatus (yellow); hm, hyomandibula; mc, Meckel’s cartilage (lower jaw); nc, nasal capsule; pop, postorbital process; pq, palatoquadrate (upper jaw); rp, rostral process; sc, spinal column; ulc, upper labial cartilage.

Beneath the chondrocranium is the visceral skeleton, which includes the upper and lower jaws, hyoid and gill arch cartilages that support the breathing and feeding structures of the head (figure 7a*–*d). The mandibular arch (first visceral arch) forms the jaw and is divided into two parts, the dorsal palatoquadrate (upper jaw) and the ventral Meckel’s cartilage (lower jaw). The hyoid arch (second visceral arch) is posterior to and closely associated with the mandibular arch. It is divided into dorsal (hyomandibula) and ventral (ceratohyal and basihyal) components. The dental structure of the rig comprises numerous small, blunt teeth aligned in both rows (vertical to the jawline, including functional teeth and their replacements) and series (parallel to the jawline) (figure 7e–h). An elongated protuberance (peg) extends from the lingual face of the crown (figure 7f). The peg of one tooth extends into the basal groove of the adjacent tooth in the same row, creating an arrangement that may serve to interlock the teeth in a pavement dentition [41]. Each tooth has a low crown with two weak, lateral cusplets (figure 7f). There are several short ridges at the base of the crown (basal ridges), characteristically found in juveniles (figure 7f,h).

## 4. Discussion

All 10 rigs (*Mustelus lenticulatus*) tested in this study produced broadband clicking sounds during handling. To the best of our knowledge, this study would be the first to show that sharks can produce sounds. Rigs handled for 20 s produced significantly more clicks during the first 10 s than during the subsequent 10 s, and both in the presence and absence of body movements. Rigs were not observed to produce clicks during feeding or while free-swimming in the tank. This may suggest that the initial handling triggers a stress or startle response, resulting in increased click activity. As rigs become accustomed to the handling, the behavioural response likely diminishes, leading to fewer clicks over time. Further behavioural observations are needed to test this hypothesis and verify whether rigs produce clicks under more natural conditions without human interference. Some species of teleost fish make clicking sounds during agonistic interactions [10,42]. For instance, conspicuous click sounds of similar intensity to those produced by the rig (mean SPL_rms_ = 156 ± 7 s.d. dB re 1 µPa at approx. 30 cm) were recorded from cod (*Gadus morhua*) in the presence of seals and human divers (mean SPL = 153 ± 7 dB re 1 µPa s.d. at 1 m) [43]. While the mechanism and purpose of the cod clicks remain unknown, behavioural observations of the nearby seals suggest they may serve a predator-startling function [43].

The first evidence of active sound production in an elasmobranch (table 2) was published by Fish & Mowbray [21] who recorded sharp clicks (peak frequency range = 1−3 kHz) from a captive cownose ray (*Rhinoptera bonasus*) following mechanical prodding [21]. The clicks were accompanied by disturbance and evasive behaviours, such as tightly closed mouth, elevated tail and raised serrate barbs [21]. It was suspected that the flat teeth were used for producing the sound based on their pavement-like morphology. Like the rig, cownose rays have flattened teeth that are arranged in upper and lower mosaic-like plates for cracking open shells of crustaceans and molluscs [44]. Recent field observations in Indonesia and Australia revealed that two stingray species, the mangrove whipray (*Urogymnus granulatus*) and the cowtail stingray (*Pastinachus ater*), produce short, broadband clicks in response to the approach of divers, likely as a behavioural reaction to stress [45] or a perceived threat [18]. The duration (range = 21−91 ms) and bandwidth (range = 22.3−23.9 kHz) of the clicks are very similar to the duration (range = 10.4−137.7 ms) and bandwidth (range = 22−24 kHz) of the rig clicks. However, the peak frequencies of the ray clicks (range = 1.03−1.88 kHz) were lower than those of the rig (range = 2.4−18.5 kHz). In both ray species clicks occurred with visible movements of the spiracle [18], an external opening near the eye in elasmobranchs that connects to the throat, via a passage between the hyomandibula and cranium [46]. It is unclear whether spiracle movement is involved in the production of click sounds in these rays. The spiracle lacks any hard skeletal structures, casting doubt on its ability to produce high-frequency sounds. Instead, its movement could result from rapid mouth closure, which forces the buccal water volume to exit through the spiracles and likely through the gill slits, although the gills are not visible in the videos [18]. Recently, three batoid species in the Mediterranean Sea—the blonde ray (*Raja brachyura*) [19], the rough skate (*Raja radula*) and the marbled electric ray (*Torpedo marmorata*) [20]—were reported to produce series of short, broadband clicks when disturbed by divers. Their clicks exhibited similar peak frequencies (range = 0.2−19 kHz, mean = 3.2–8.4 kHz) to those produced by the rig (range = 2.4–18.5 kHz, mean = 9.6 kHz). In the marbled electric ray, sound production was accompanied by synchronized opening and closing of the mouth and movements of the pectoral fins, suggesting the sound might originate from the oral cavity [20]. Similarly, coordinated pectoral fin movements with click production were observed in the rough skate [20]. In the present study, rigs were submerged during handling, providing only a dorsal view of the shark, which made it impossible to observe small-scale movements of the spiracle or lower jaw during handling. Clearly, more investigations, using close-up underwater video recordings are needed to determine which body parts are involved in the generation of clicks in both rays and the rig.

**Table 2.** Documented cases of active sound production^a^ in elasmobranchs.

species (order)	sound type	peak frequency (kHz) range (mean ± s.e.)	mean bandwidth (kHz)	duration (ms) range (mean ± s.e.)	period (s) range (mean ± s.e)	behavioural context	body parts proposed for sound producing mechanism
*Rhinoptera bonasus*^b^ cownose ray (Myliobatiformes)	click	1−3	—	—	—	forceful prodding, agonistic display	flattened teeth
*Pastinachus ater*^c^ cowtail stingray (Myliobatiformes)	click	1.4−1.5	23.9	21−91 (65 ± 12)	—	approaching diver, subsequent fleeing	contraction of spiracles and cranial area
*Urogymnus granulatus*^c^ mangrove whipray (Myliobatiformes)	click	1.03−1.5 1.69−1.88	22.73 22.31	17−25 (21 ± 1) 10−17 (13 ± 1)	—	approaching diver	contraction of spiracles and cranial area
*Raja brachyura*^d^ blonde ray (Rajiformes)	click	6.28−7.22 (6.93 ± 0.08)	—	10−20 (15 ± 0.9)	0.5−0.83 (0.58 ± 0.03)	startled by camera, subsequent fleeing	—
*Raja radula*^e^ *rough skate* *(Rajiformes)*	click	0.2−19 (3.15 ± 1.1)	21.28	25−82 (24 ± 3.4)	0.43−0.81 (0.57 ± 0.03)	approaching diver	opening and closing of mouth, movement of pectoral fins
*Torpedo marmorata*^e^ marbled electric ray (Torpediniformes)	click	0.12−17.7 (8.39 ± 0.95)	17.37	4−16 (9.3 ± 0.6)	0.54−0.78	approaching diver, agonistic display	movement of pectoral fins
*Mustelus lenticulatus*^f^ rig (Carcharhiniformes)	click	2.44−18.5 (9.56 ± 0.34)	23	10.4−137.7 (48.4 ± 2.9)	0.019−9.57 (1.42 ± 0.16)	hand-held	flattened teeth

^a^ Active sound production is defined as the intentional generation of sound by the animal via an internal mechanism [6,21]. By contrast, passive sounds are produced unintentionally and co-occur as by-products of specific activities, such as swimming (e.g. veering, struggling, breaching), aggressive encounters (e.g. collision), manipulation of prey and feeding (e.g. shell crushing, chewing).

^b^ Fish & Mowbray [21].

^c^ Fetterplace *et al*. [18].

^d^ Barroil *et al*. [19].

^e^ Almagro & Barría [20].

^f^ Present study.

Sounds produced by stridulation (based on friction of skeletal elements such as teeth, fin rays and vertebrae) generally have a wide bandwidth and the maximum spectral output is usually located above 1 kHz to up to several kHz [3,14,34]. In this regard, the broadband clicks exhibited by the rig have a frequency range that is characteristic of stridulatory sounds. Stridulation requires two hard structures that provide a surface where friction can occur. The morphological examinations of the cranial structures in the rig conducted here did not reveal any obvious specialized sound producing structures apart from the teeth that could be responsible for producing these clicks. Our study notes that the denture apparatus in the rig is similar in shape throughout the upper and lower jaw (homodont dentition) and is composed of blunt, fattened teeth that form hard, pavement mosaic structures [47]. The shape and arrangement of the teeth are linked to their ability to crush prey, primarily crustaceans [48]. This tooth morphology is similar to that found in skates and rays [44]. We suggest that this teeth arrangement could also be linked to sound production via a snapping mechanism.

Numerous species of teleosts use dental friction (e.g. grinding or snapping) to produce distress and aggression sounds [49–51]. The teeth of teleost fish are characterized by long roots that are firmly cemented deep within the jawbone (thecodont dentition). By contrast, the teeth of elasmobranchs are more loosely embedded in the dermis and connected by less rigid connective tissues [52]. This lack of firmness in tooth arrangement may make it less likely for stridulation to occur through grinding, as this mechanism requires structures that resist imposed movements. However, the tooth pegs (elongated protuberances) in *Mustelus* may serve to interlock the teeth to form a firm surface [41], which could facilitate teeth grinding. In the case of teeth grinding, sounds generally manifest as a longer series of pulses where the pulses are generated by a series of frictions [49]. As in the rays [18–20] the rig click sounds are relatively short (mean = 48 ms), which is more consistent with a snapping mechanism. Within the limits of the available data, the broadband frequency range and short duration of the rig clicks suggest the involvement of teeth snapped during rapid mouth closure for sound production. However, additional investigations will be necessary to test this hypothesis.

Given the shared pavement-like dentition among all species of the genus *Mustelus* [48], it is likely that other *Mustelus* species produce similar sounds. We therefore conducted initial tests with a species from the western Atlantic Ocean, namely the dusky smoothhound (*M. canis*) in October 2024 at the Woods Hole Oceanographic Institution, MA, USA (approved by the WHOI Institutional Animal Care and Use Committee protocol BI30464.00). Similar to the rig, the dusky smoothhound is an opportunistic benthic predator that prefers crustaceans, particularly crabs and shrimps [53], which it crushes with its flattened teeth [48]. In a manner similar to our experiments with the rig, three dusky smoothhounds (TL range = 55−78 cm) were hand-held underwater in a holding tank for 20 s and monitored for sound production. However, none of the three dusky smoothhounds produced any sounds during handling. Notably, the three dusky smoothhounds had been in captivity for about four months before testing, possibly habituating to human interactions. Our observations align with Fish & Mowbray [21], who auditioned a captive dusky smoothhound through handling and mechanical prodding but did not detect any biological sounds produced by this species. The smoothhound shark genus *Mustelus* (family Triakidae) comprises 27 species of small (0.6–1.85 m) mesopredatory sharks, showing a high degree of regional endemicity [28,48,54]. These benthopelagic sharks are abundant worldwide in temperate to tropical coastal habitats from shallow waters to a maximum depth of approximately 1500 m [28,41]. There are two *Mustelus* clades, one comprising species with no white spots and a placental reproductive mode (placental clade) and a second subgroup of aplacental species with white spots (aplacental clade) [55], which evolved secondarily from the placental species [56]. The dusky smoothhound (western Atlantic) belongs to the placental, unspotted clade and the rig (endemic to New Zealand Pacific) to the aplacental, white spotted clade [55]. Besides their differences in reproductive modes and geographical distribution, the two species may have also evolved distinct behavioural strategies in response to disturbances, such as click production in the rig but not in the dusky smoothhound.

Whether rigs can acoustically sense their click sounds is not yet understood. Auditory evoked potential measurements revealed that the rig is most sensitive to very low frequencies, around 150 Hz and has an upper frequency limit at 800 Hz [57,58]. The main energy of the clicks is centred within 2.4−18.5 kHz, which exceeds the upper frequency detection limit of this species and casts doubt on any role in conspecific communication. However, the initial impulse exceeds sound pressure levels (rms) of 160 dB re. 1 µPa (at approx. 30 cm) and contains low frequencies (<1 kHz). Thus, there might be the possibility that rigs can acoustically detect the clicks. Complex acoustics in experimental tanks, due to reverberation, reflection and resonance, can cause signal distortion [23,40,59]. For instance, tanks can attenuate low frequencies [60], and reverberations can make sounds last longer, leading to potential overestimation of signal duration [40,61]. Future studies should attempt to record the clicks in an unbound medium in terms of both sound pressure and particle acceleration, the latter being the main acoustic signal detected by sharks [62,63].

The purpose of the clicks remains uncertain; they could be either an incidental (physiological, mechanical) byproduct of a startle response or a meaningful biological signal (e.g. alarm, warning, defence). For instance, clicks could represent a ‘predator-related’ sound [10]. Rigs are vulnerable to predation and are often consumed by larger predators, such as toothed whales, larger sharks and teleosts [30,31,64–66]. Apart from the initial impulse (with energy below 1 kHz) most of the click’s energy is outside the auditory range of most predatory fishes [65], but well within the hearing ranges of many predatory marine mammals [66]. However, marine mammal predators may not be deterred from attacking a small shark due to clicks. In fact, many teleosts have been observed to emit ‘predator-related’ sounds when predators approach or when being caught [10]. But none of the predators investigated so far responded to these calls [4,10]. Further investigations are needed to determine whether rigs produce clicks in natural behavioural contexts and, if so, under which conditions (e.g. predator threats, attacks or agonistic encounters).

## 5. Conclusions

This study showed that rigs produce broadband clicks when handled underwater, representing the first documented case of active sound production by a shark. The relatively high peak frequencies and short duration of the sounds, and the plated teeth morphology suggest that forceful snapping of the teeth is the likely sound producing mechanism, but further studies are necessary to confirm this hypothesis. Our observations suggest that rigs produce these sounds in response to disturbance or distress. Future behavioural studies are warranted to address as to whether clicks represent a biological meaningful signal.

## Data Availability

Data are available as electronic supplementary material [67].
